# Cost-effectiveness of preimplantation genetic testing for aneuploidy for women with subfertility in China: an economic evaluation using evidence from the CESE-PGS trial

**DOI:** 10.1186/s12884-023-05563-z

**Published:** 2023-04-14

**Authors:** Xuan He, Xiao Wang, Jiaojie Shen, Bin Wan, Yingpeng Wang, Zhuolin Zhang, Lele Cai, Yuwen Bao, Haixia Ding, Xin Li

**Affiliations:** 1 School of Health Policy and Management, Nanjing Medical University, Nanjing, China; 2 School of Pharmacy, Nanjing Medical University, Nanjing, China; 3 Department of Health Insurance Management, The First Affiliated Hospital with Nanjing Medical University, Nanjing, China; 4 Center for Global Health, School of Public Health, Nanjing Medical University, Nanjing, China

**Keywords:** In vitro fertilization, Preimplantation genetic testing, Aneuploidy, Cost-effectiveness, CESE-PGS trial

## Abstract

**Background:**

There are a large number of infertile couples in China, but its treatment is notoriously expensive and not currently covered by insurance. The utility of preimplantation genetic testing for aneuploidy as an adjunct to in vitro fertilization has been debated.

**Objective:**

To investigate the cost-effectiveness of preimplantation genetic testing for aneuploidy (PGT-A) versus conventional technology in in vitro fertilization (IVF) from the perspective of the healthcare system in China.

**Methods:**

Following the exact steps in the IVF protocol, a decision tree model was developed, based on the data from the CESE-PGS trial and using cost scenarios for IVF in China. The scenarios were compared for costs per patient and cost-effectiveness. One-way sensitivity analysis and probabilistic sensitivity analysis were performed to confirm the robustness of the findings.

**Main outcome measures:**

Costs per live birth, Costs per patient, Incremental cost-effectiveness for miscarriage prevention.

**Results:**

The average costs per live birth of PGT-A were estimated as ¥39230.71, which is about 16.8% higher than that of the conventional treatment. Threshold analysis revealed that PGT-A would need to increase the pregnancy rate of 26.24–98.24% or a cost reduction of ¥4649.29 to ¥1350.71 to achieve the same cost-effectiveness. The incremental costs per prevented miscarriage was approximately ¥45600.23. The incremental cost-effectiveness for miscarriage prevention showed that the willingness to pay would be ¥43422.60 for PGT-A to be cost-effective.

**Conclusion:**

The present cost-effectiveness analysis demonstrates that embryo selection with PGT‑A is not suitable for routine applications from the perspective of healthcare providers in China, given the cumulative live birth rate and the high costs of PGT‑A.

**Supplementary Information:**

The online version contains supplementary material available at 10.1186/s12884-023-05563-z.

## Introduction

Aneuploidy is common among preimplantation human embryos used in in vitro fertilization (IVF). Abnormal chromosome numbers can negatively affect the outcome of IVF such as implantation failure or miscarriage [1]. To maximize the success of IVF, various techniques have been employed to aid the selection of the embryo. The conventional morphological analysis does not prevent aneuploid embryos from being transferred [2].

Preimplantation genetic testing for aneuploidies (PGT-A) provides a robust alternative to avoid the selection of embryos with aneuploidy [3–6]. PGT-A may improve the outcome of IVF, potentially shorten the time to pregnancy and decrease the miscarriage rate [7, 8]. As the technology for genetic testing continues to improve, so will the accuracy and effectiveness of PGT-A [9]. However, due to the traumatic nature of PGT-A and the existence of chimerism problems, [10] PGT-A has certain indications and is more used in elderly patients and patients with poor pregnancy history in China. The question of whether PGT-A improves IVF outcomes or not has been debated in the literature [11–14].

Patients may perceive the use of PGT-A as an effective way of excluding abnormal embryos, thus helping to reduce unnecessary embryo transfers and decrease the cost [15]. In the absence of definitive clinical guidance, the physical, mental, and financial burdens may be important deciding factors when a patient chooses PGT-A. Although it is difficult to quantify, as cycle costs vary considerably, the cost-effectiveness of PGT-A is an increasingly critical issue [16]. Some previous studies argued that it could be cost-effective in specific clinical settings and population groups [17–20]. Conversely, other studies revealed that PGT‑A could not be recommended from a cost-effectiveness perspective [21–23]. To date, there is no published study reporting on the cost-effectiveness of PGT-A in China.

Of note, a largest randomized multicenter clinical study (CESE-PGS trial) of aneuploidy has discovered that the cumulative live birth rate (CLBR) for women between 20 and 37 year-old has not been effectively improved in China. Nevertheless, due to PGT-A, fewer embryo transfers were required, and fewer miscarriages occurred to achieve the same CLBR compared with the control group [24].

Currently, subfertility patients are required to bear 100% of the costs of assisted reproductive treatments themselves in most areas of China. On February 21, 2022, the Beijing Health Insurance Bureau released a basic health insurance reimbursement policy for assisted reproductive technology, including 16 programs, and it would officially take effect on March 26, 2022 [25]. The policy covered both preimplantation genetic testing for monogenic/single gene (PGT-M) and structural chromosome rearrangements (PGT-SR), but PGT-A was excluded.

Information on the cost-effectiveness of PGT-A for women undergoing IVF can help patients, clinicians, and health insurers in their decisions making process regarding to whether utilizing and paying for new technology or not. This study aimed to examine the cost-effectiveness of PGT‑A in women with a good prognosis from the Chinese healthcare system perspective.

## Materials and methods

**Fig. 1:**
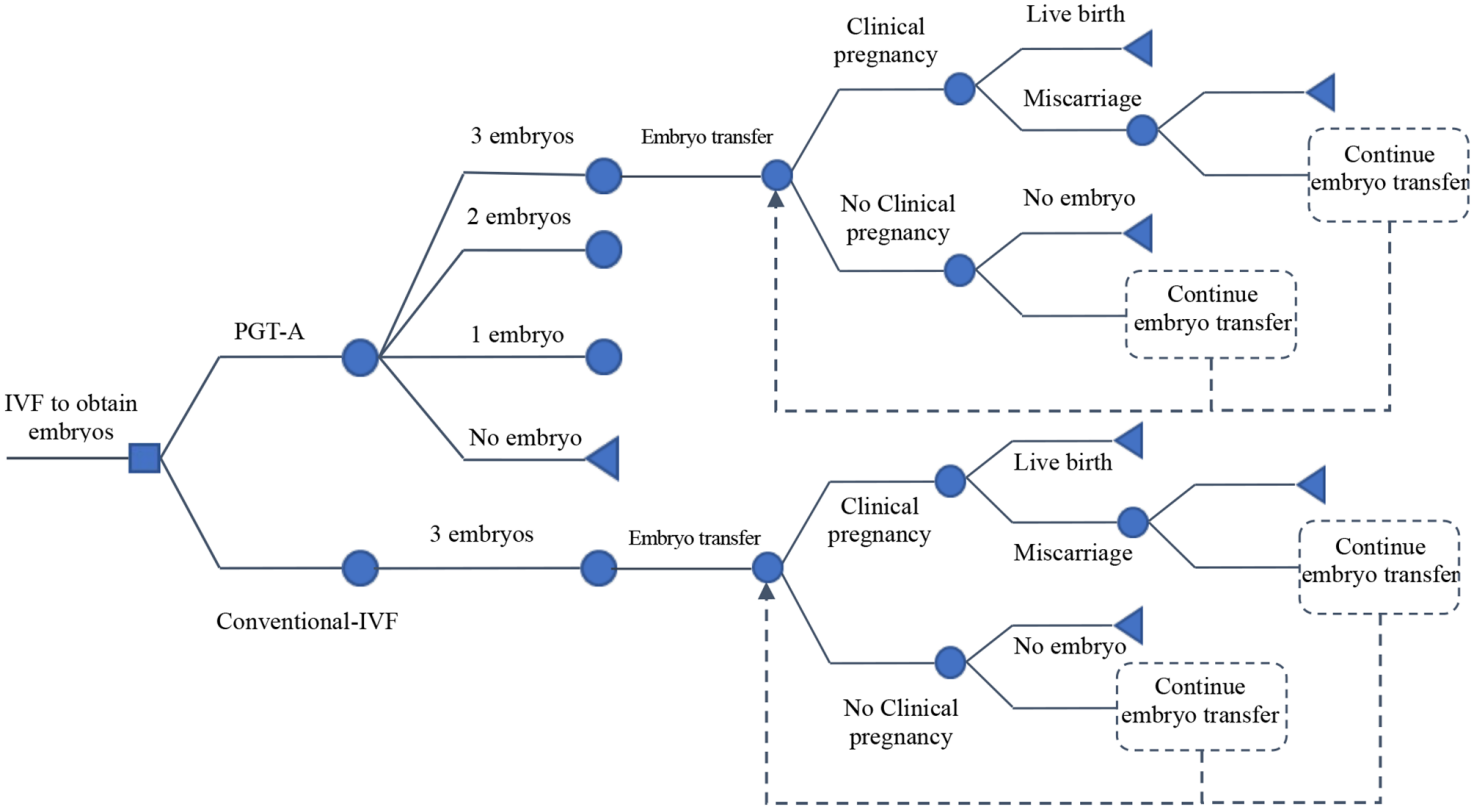
Decision tree model based on the CESE-PGS trial. Note: IVF, In Vitro Fertilization; PGT-A, Preimplantation genetic testing for Aneuploidy. As indicated in Fig. 1, one embryo refers to one subsequent embryo transfer cycle initiated, two embryos refer to two subsequent embryo transfer cycles initiated, and so on. The “+” stands for the same decision structure as above. Nodes within the model are marked by circles, triangles define endpoints

A decision tree model based on data from the CESE-PGS trial was developed using the TreeAge Pro Suite 2011 software (TreeAge Software, Inc., Williamstown, MA, USA) (Fig. 1). The decision tree is a decision-making model that simulates a group of patients following a predefined approach with relevant probability, cost, and result [26]. Two treatment strategies were modeled: IVF with PGT-A versus IVF without PGT-A. As no human participants were involved in this theoretical analysis, this study was exempt from approval from the institutional review board. This economic evaluation used no individual patient-level data to inform the model. This study was conducted and reported following the Consolidated Health Economic Evaluation Reporting Standards (CHEERS) reporting guideline [27].

The model incorporated main clinical events that were critical and would incur costs were chosen including PGT-A, blastocyst transfer, clinical pregnancy, miscarriage, and live birth. For both groups, each transfer had 3 distinct possible outcomes: not pregnant, clinical miscarriage, and live birth. The analysis of the decision tree model allowed us to obtain the probability of pregnancy, miscarriage, and live birth for the patient in all cases. Any pregnancies that do not result in a live birth, such as a pregnancy termination or an ectopic pregnancy, were classified as a clinical miscarriage.

There were several key assumptions in the model. In this model, the patients who underwent assisted reproduction treatment were simulated analogously to the CESE-PGS trial. Patients in both treatment groups underwent one cycle of controlled ovarian stimulation, followed by IVF with PGT-A or IVF with no selection by genetic status and transfer of a maximum of three selected embryos. Intracytoplasmic sperm injection (ICSI) was used in all IVF procedures. In the PGT-A group, three good-quality blastocysts that had been selected by using morphologic criteria underwent trophectoderm biopsy based on the next-generation sequencing platform (Illumina Next Seq 550 or Ion PGM/Proton). For safety reasons, only euploid blastocyst was chosen for transfer in the PGT-A group. All the obtained embryos were cryopreserved and single frozen-embryo transfers were performed each time in the two groups.

The main inclusion criteria of the CESE-PGS trial were as follows: eligible patients were 20–37 years old and the availability of three or more good-quality blastocysts. Exclusion criteria as a known uterine abnormality, the presence of a contraindication to live birth, a plan to undergo PGT-M or PGT-SR, or the use of donated sperm or oocytes to achieve live birth. We characterize the population to which our results may be generalized (eTable 1 in the Supplement).

## Effectiveness

The Primary outcome/effectiveness of the model was the cumulative live birth rate (CLBR), the sum of the probability of live birth after all embryo transfers were completed, and secondary effectiveness was the cumulative miscarriage rate (CMR), the sum of the probability of miscarriage among all embryo transfers completed.

## Probability

The probability inputs for clinical outcomes were estimated from a PGT-A study, which is currently the largest, multicenter, randomized, and controlled study in China [24]. The study included 14 academic fertility centers throughout China, which is more representative of China’s population and made the findings more generalizable than that from single-center analyses. Probabilities were estimated from the published absolute frequencies as several events per total number satisfying the condition.

The probability of clinical pregnancy was calculated as the average clinical pregnancy over all embryo transfer cycles. We generated estimates of probabilities of live birth or miscarriage per clinical pregnancy and estimates of probabilities of clinical pregnancy per embryo transfer.

For the PGT-A group, there were 4 different possible scenarios following the test: 3 embryos available for transfer, 2 embryos available for transfer, 1 embryo available for transfer, and no embryos available for transfer. The probability of each scenario was calculated based on the probability of balanced euploid from the result of preimplantation genetic testing.

All probabilities used in the study are shown in Table 1.

**Table 1:** Input Parameters in the base case and distributions of sensitivity analysis

Variable	Distribution	One-way sensitivity analysis			Expected value	Parameters	Source
		Min		Max			
**Cost, CNY**							
PGT-A (3 embryos)	log-normal	0	10,000		6,000	(u = 8.70, s = 0.32)	Local data
IVF procedures in IVF stage ^a^	log-normal	6,375	10,625		8,500	(u = 9.04, s = 0.32)	Local data
Examination in IVF stage ^a^	log-normal	1,125	1,875		1,500	(u = 7.31, s = 0.32)	Local data
Drugs in IVF stage ^a^	log-normal	2,850	4,750		3,800	(u = 8.24, s = 0.32)	Local data
IVF procedures in ET stage ^b^	log-normal	3,450	5,750		4,600	(u = 8.43, s = 0.32)	Local data
Examination in ET stage ^b^	log-normal	375	625		500	(u = 6.21, s = 0.32)	Local data
Drugs in ET stage ^b^	log-normal	1,500	2,500		2,000	(u = 7.60, s = 0.32)	Local data
Miscarriage	log-normal	1,500	2,500		2,000	(u = 7.60, s = 0.32)	Local data
Live birth	log-normal	3,000	5,000		4,000	(u = 8.29, s = 0.32)	Local data
**Probability**							
Clinical pregnancy, PGT-A Group	Beta ^c^	0.5400	0.9000		0.7200	(α = 504, β = 196)	J. Yan et al., 2021
Clinical pregnancy, Conventional-IVF Group	Beta ^c^	0.5156	0.8593		0.6874	(α = 574, β = 261)	
Live birth after clinical pregnancy, PGT-A Group	Beta ^c^	0.6815	1.0000		0.9087	(α = 458, β = 46)	
Live birth after clinical pregnancy, Conventional-IVF Group	Beta ^c^	0.6455	1.0000		0.8606	(α = 494, β = 80)	

Note: IVF, In Vitro Fertilization; ET, embryo transfer

^a^ IVF stage includes all procedures from the patient’s subfertility diagnosis to the acquisition of embryos;

^b^ ET stage includes all procedures from embryo transfer to live birth;

^c^ Parameters are numbers with and without the feature for beta distributions

## The scenarios of cost

The research perspective of the study was that of the healthcare system and non-medical costs and costs of time and loss of productivity were not incorporated in the analysis. Costs were indexed to 2021, and no discounting was applied due to the short time horizon employed.

The scenarios reflected the direct medical cost of IVF for subfertility treatment in China. We estimated the cost of IVF separately based upon the local charges on an out-of-pocket basis.

The costs of IVF for patients were from a tertiary public hospital, which was one of 14 centers in the CESE-PGS trial, located in Jiangsu Province, China from 2019 to 2021.

## Cost assessment

The direct medical costs primarily included examination costs before patients underwent the subfertility treatment and IVF-related costs during the entire cycle. Based on expert research and analysis of medical costs in hospital information systems, the direct medical costs of IVF were divided into examination costs including laboratory costs and ultrasound procedures costs, drugs costs, and IVF procedures costs. The drug costs were assumed to include the costs of controlled ovarian hyperstimulation, endometrial preparation, and luteal-phase support. The costs of IVF procedures were estimated to cover the costs of ovum pickup, IVF laboratory procedure, cryo-preserved embryo transfer, and thawing procedure. Calculation of the costs incurred by PGT‑A included the cost of performing genetic analysis of three blastocysts (eTable 2 in the Supplement).

We also collected direct medical costs associated with patients’ miscarriages and live births to estimate costs. The costs of Miscarriage were assumed to include dilation and curettage with anesthesia. The costs of prenatal checkups and cesarean sections belonged to the cost of live birth. In this study, only direct medical costs were used in the assumption analysis, as indirect costs were highly variable and difficult to calculate. All incorporated costs (in CNY) are shown in Table 1.

### Cost-effectiveness

The cost-effectiveness and outcome were expressed by ratio using the following formula:

$$\begin{array}{*{20}{l}}{{\rm{Costs}}\,{\rm{per}}\,{\rm{live}}\,{\rm{birth}}\left( {{\rm{Cost - effectiveness}}} \right){\rm{ = }}}\\{{\rm{Average}}\,{\rm{costs}}\,{\rm{per}}\,{\rm{patient/CLBR}}}\end{array}$$

$$\begin{array}{*{20}{l}}{{\rm{Average}}\,{\rm{costs}}\,{\rm{per}}\,{\rm{patient = }}}\\{\Sigma \,{\rm{Path}}\,{\rm{cost}}\,{\rm{*}}\,{\rm{Path}}\,{\rm{probability}}}\end{array}$$

The incremental cost-effectiveness ratio (ICER) for live birth was defined as the difference in cost divided by the difference in cumulative live birth rate (CLBR). Similarly, the incremental cost-effectiveness ratio (ICER) for miscarriage prevention was defined as the difference in cost divided by the difference in cumulative miscarriage rate (CMR).

$$\begin{array}{*{20}{l}}{{\rm{ICER}}\,{\rm{for}}\,{\rm{live}}\,{\rm{birth}} = }\\{{\mkern 1mu} {\mkern 1mu} {\mkern 1mu} {\mkern 1mu} {\mkern 1mu} {\mkern 1mu} {\mkern 1mu} {\mkern 1mu} \frac{{COS{T_{PGT - A\,group}} - COS{T_{conventional\,IVF\,group}}}}{{CLB{R_{PGT - A\,group}} - CLB{R_{conventional\,IVF\,group}}}}}\end{array}$$

$$\begin{array}{*{20}{l}}{{\rm{ICER}}\,{\rm{for}}\,{\rm{miscarriage}}\,{\rm{prevention}} = }\\{{\mkern 1mu} {\mkern 1mu} {\mkern 1mu} {\mkern 1mu} \frac{{COS{T_{PGT - A\,group}} - COS{T_{conventional\,IVF\,group}}}}{{CM{R_{PGT - A\,group}} - CM{R_{conventional\,IVF\,group}}}}}\end{array}$$

### Sensitivity analysis

The one-way sensitivity analysis was conducted to ensure the robustness of the results. A threshold analysis of the maximum tolerable cost of PGT-A was carried out, which would still provide the same cost per live birth as the conventional-IVF group. In addition, the theoretical threshold for the clinical pregnancy rate to achieve the cost-effectiveness of PGT-A was estimated. One-way sensitivity analysis was performed by changing a parameter every time to the highest or lowest values. The expenditures were set to fluctuate between − 25% and + 25%. The theoretical necessary thresholds for the maximum cost of PGT-A and the clinical pregnancy rate to achieve cost-effectiveness of PGT-A were also estimated. The cost of PGT-A varied in the range of ¥0–10 000. A Tornado graph was used to show the influential variables and the results of one-way sensitivity.

The probabilistic sensitivity analysis was conducted by varying all parameters within a set of different distributions simultaneously in 1000 Monte Carlo simulation iterations to illustrate the results of uncertain analysis and build a cost-effectiveness acceptability curve [28]. To conduct the analysis, effects were replaced by beta distributions and costs by lognormal distributions. Beta distributions were assumed for probabilities, and their parameters were based on the figures observed in the CESE-PGS trial. Log-normal distributions were for costs, with assumed median values based on the specifications for the respective base-case scenarios. All distributions used are shown in Table 1.

## Results

### Costs per live birth and average costs per patient

Compared with the conventional-IVF group, the costs of the PGT-A group were higher, with a lower CLBR and a lower CMR. The PGT‑A significantly increased the costs per live birth. As shown in eFigure 1 in the Supplement, the average costs per live birth were ¥33587.65 for the conventional-IVF group and ¥39230.71 for the PGT-A group. In line with the increased costs per live birth, the average costs per patient was also increased for patients undergoing PGT-A, in a word, if PGT‑A was carried out, the average costs per patient was about 9.23% higher than that of the conventional IVF.

### The incremental costs of miscarriage prevention

The average costs per patient in both groups in the model for miscarriage prevention was the same as the results of the model using live birth as an indicator. However, compared with the conventional-IVF group, the CMR in the PGT-A group was lower, resulting in an ICER of ¥45600.23 to prevent one miscarriage.

### Sensitivity analysis

#### One-way sensitivity analysis

**Fig. 2:**
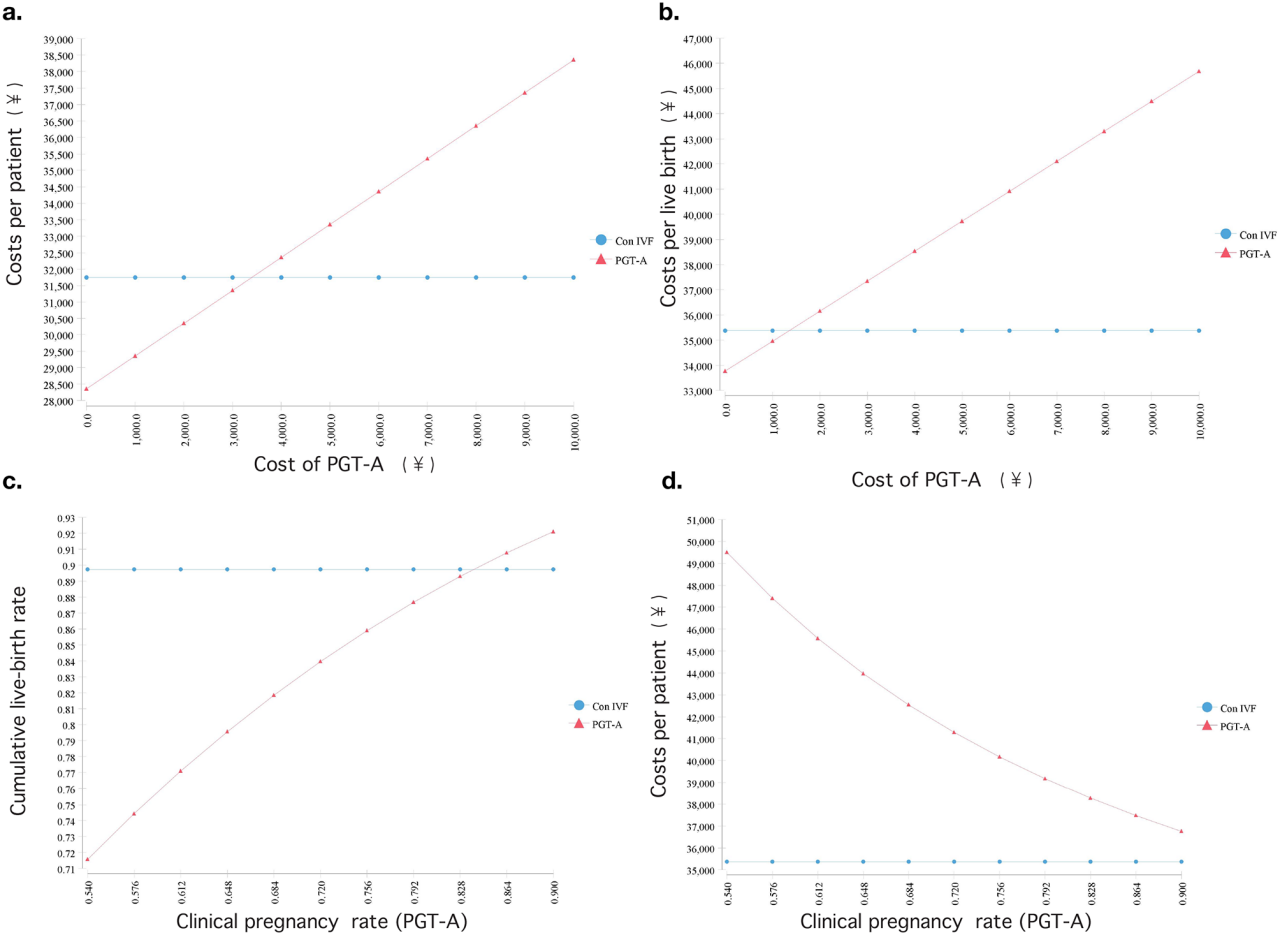
One-way sensitivity analysis of cost of PGT-A and Clinical pregnancy rate of PGT-A. Note: Con IVF, conventional-IVF; PGT-A, preimplantation genetic testing for aneuploidy. One-way sensitivity analysis for the dependence of the costs per patient (**a**), per live birth (**b**) on the cost of PGT-A. One-way sensitivity analysis for the dependence of the CLBR (**c**) and per patient (**d**) on the Clinical pregnancy rate of PGT-A

One-way sensitivity analysis showed that the costs of PGT-A would have to be at least ¥3393.95 to achieve the same costs per patient as the conventional-IVF group. The costs of PGT-A were continuously adjusted in the threshold analysis, and in this simulation, PGT‑A became cost-effective compared to the conventional-IVF group when the costs of PGT‑A was ¥ 1350.71 or less. When the clinical pregnancy rate in the PGT-A group increased to 83.88%, patients had the same CLBR as in the conventional-IVF group, and a clinical pregnancy rate of 98.24% would be required to achieve the same costs per live birth (Fig. 2).

**Fig. 3:**
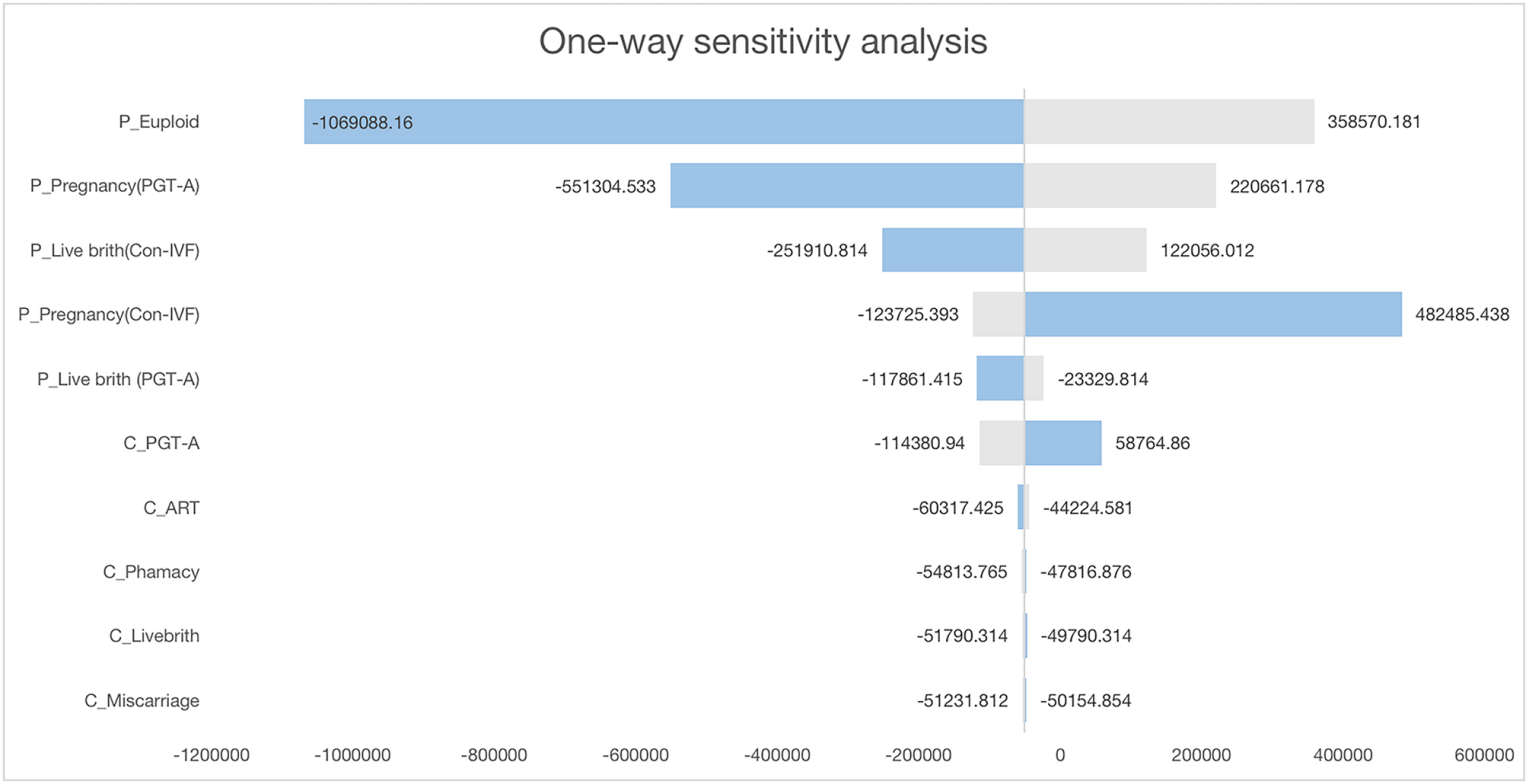
Tornado diagram. Note: The parameters affecting the ICER are shown in above figure

The tornado diagram of one-way sensitivity analysis showed that the ICERs in descending order were sensitive to which parameters (Fig. 3). The factors that had a greater impact on the total outcome were the probability of euploid and clinical pregnancy. The results showed that this model was relatively robust.

#### Probabilistic sensitivity analysis (PSA)

**Fig. 4:**
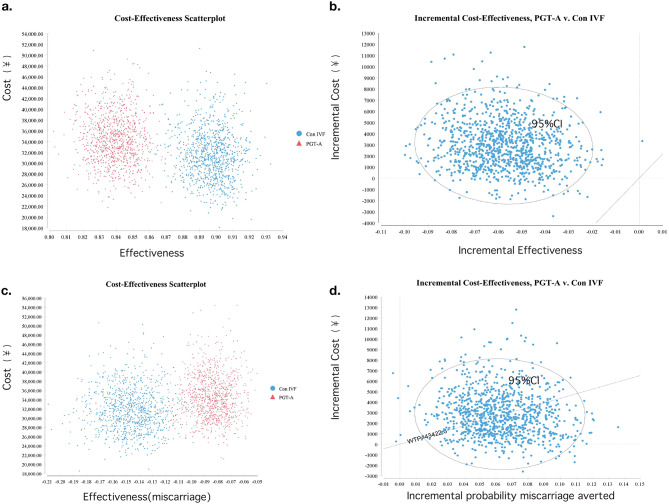
Cost-Effectiveness Plane of probabilistic sensitivity analysis between conventional-IVF and PGT-A. Note: (**a**) showing the cost-effectiveness with live birth as effectiveness between conventional-IVF and PGT-A, (**b**) showing the ICERs of PGT-A to increase the probability of live birth, (**c**) showing the cost-effectiveness with miscarriage as effectiveness between conventional-IVF and PGT-A, (**d**) showing the ICERs of PGT-A to lower probability of miscarriage

A PSA showed cost-effectiveness for costs per live birth for PGT-A for 0%. Further results of the PSA are shown in eTable 3 in the Supplement. The incremental cost-effectiveness for miscarriage prevention showed that the willingness to pay would be ¥43422.60. Cost-effectiveness acceptability curves are shown in eFigure 2 in the Supplement. The cost-effectiveness and Incremental cost-effectiveness scatter plots are shown is in Fig. 4.

## Discussion

### Main findings

To our knowledge, this is the first study that revealed the cost-effectiveness of PGT-A in the Chinese healthcare system settings. This study showed that patients aged 20 to 37 years of old with fertility problems undergoing PGT-A (with cryopreservation and subsequent frozen embryo transfer cycles) contributed to an average of ¥ 5643.06 more per live birth. The addition of PGT‑A led to an increase in the costs of IVF treatment.

More women in the conventional-IVF group underwent a second or third embryo-transfer cycle. Thus, the average costs of the two groups are not as different as expected. However, this differential did not translate into a higher CLBR and less direct medical costs for patients. From the healthcare system’s perspective, the findings of this cost-effectiveness analysis showed that PGT‑A could significantly lead to higher costs per patient. With the CLBR as the primary goal, conventional-IVF in infertile patients aged 20 to 37 years of age was the optimal treatment strategy.

According to the annual report of assisted reproductive technology of the Chinese Society of Reproductive Medicine [29, 30], the application of PGT-A in China has steadily increased, and its clinical pregnancy rate has increased significantly. The ART data system reported 3 580 (0.79%) PGT-A cycles in 2018. The pregnancy rate and live birth rate of PGT-A cycles in 2019 were 58.32% and 48.35%, respectively, which were significantly higher than those of conventional-IVF cycles. With the development and promotion of PGT-A in China, it is foreseeable that there will be more applications of PGT-A. Recent technological development has already brought cost reduction to PGT-A. However, a threshold analysis of the maximum costs of a PGT‑A which would result in the same costs per live birth as in the control group showed results that were significantly less than the costs of PGT-A for 3 oocytes. At the same time, not all institutions have the capacity to provide PGT-A, which requires increasing resources and up to 8 cumulative hours of labor from the embryology team for each biopsy case [31].

In this model, we conclude that PGT-A was not a cost-effective tool to increase the number of live births in the population. We recommend the conventional-IVF as a suitable solution for patients who are not sensitive to the miscarriage cost, as this option could ensure patients meet the best cost-effectiveness. Our findings demonstrate that pursuing the advanced PGT-A functions is not related to better live birth outcomes and lower economic burden from the patients’ perspective. The findings are in line with the results of previous studies which focused on the subfertility patients with PGT-A in Germany and the USA [22, 23, 32].

On the other hand, the ICER for miscarriage prevention was ¥45600.23. It represents the prevention of one miscarriage is a rather high cost, which is beyond most patients’ willingness to pay. In the Chinese context, WTP for 1 year of life or quality-adjusted life is commonly set somewhere between 1 and 3 times GDP per capita, but that WTP range is difficult to translate. Specific WTP guidelines for miscarriage prevention remain an evidence gap in the field. The probabilistic sensitivity analysis showed that the probability of PGT-A was dominant is 50.00% for miscarriage prevention when the threshold of willingness to pay was ¥ 43422.60. There are pros and cons to calculate cost-effectiveness with the threshold of averting miscarriage or increasing live birth [32]. The CLBR has been recommended as the most relevant patient-centered outcome in clinical trials of infertility treatment [33]. Reducing miscarriage without increasing live birth misses the primary goal of treatment. It is necessary to counsel the patients on how to choose different regimens based on the chances of conceiving as well as carrying a baby to term. Only considering the risk of miscarriage of each treatment option is insufficient.

Reproductive experts and policy-makers in German believed that moderate copayment IVF technology is acceptable, and private insurance alone was not sufficient [34, 35]. Many developed countries have already established IVF health insurance mechanisms, and some developing countries, such as China, also need to explore a hybrid payment mechanism that meets the realistic demands. Although the government provides partial subsidies in some areas of China, the cost of IVF technology remains a catastrophic cost for poor families [36]. The cost-effectiveness study of IVF is important, not only in informing treatment choices for individuals and families but also in providing policy recommendations for healthcare systems and the whole society. PGT-A has not covered the IVF medical insurance policy issued by the Beijing Municipal Bureau of Medical Security, but PGT-M and PGT-SR, which are also genetic tests, are covered, which may be related to its scope of application. PGT-A has a broader population than the other two PGT techniques. In this study, it is still difficult to have cost-effectiveness for infertile women with a good prognosis. However, the cost-savings associated with PGT-A will obviously be driven by the costs of embryo transfer and miscarriage, as genetic testing technology advances decrease its cost PGT-A still has the potential to be a way to save health care costs and avoid or reduce the waste of health care resources.

### Strengths and limitations

This study has several limitations. First, It is noteworthy that many miscarriages could indeed be managed conservatively and will not require costly dilation and curettage with anesthesia [37]. We acknowledge that our analysis does not include indirect costs. It is important to emphasize that our analysis only deals with the direct medical costs of managing a miscarriage. Miscarriages may have deleterious psychological consequences which may also lead to the significant indirect cost (e.g. absence from work, further healthcare resource utilization) in addition to the anxiety and burden on the patient [38]. Second, we only include the women who had a good prognosis for live birth, among whom only three embryos were tested in the PGT-A group, and only up to three transfers in one year were included in the trial. For the patients in the same age group, higher LBR was observed in the CESE-PGS trial compared to treatment outcomes recorded in the Chinese IVF Register database [30]. Thus, in other populations with subfertility, cost-effectiveness analysis would likely show different conclusions. Third, the incidences of moderate or severe ovarian hyperstimulation syndrome, ectopic pregnancy, obstetrical or perinatal complications, and congenital anomalies were also similar in the two groups. Therefore, other adverse clinical events were not considered for the sake of model simplification.

## Conclusion

The results demonstrate that embryo selection with PGT‑A is not suitable for routine applications in view of the CLBR and the high costs of genetic testing from the theoretical frameworks in health economics in China, it also appears that PGT-A is an expensive way to reduce miscarriage without increasing the chance of achieving a live birth. Our study provides a reference for the government and policy makers to support the future inclusion of IVF treatment in health insurance payments.

## Electronic supplementary material

Below is the link to the electronic supplementary material.

**Additional file 1: eTable 1.** Characteristics of the Patients in CESE-PGS trial

**Additional file 2: eTable 2.** Procedures for incorporating cost estimates in IVF

**Additional file 3: eTable 3.** Results of probabilistic sensitivity analysis

**Additional file 4: eFigure 1.** Costs per live birth and per patient with and without PGT-A in the base-case

**Additional file 5: eFigure 2.** Cost-effectiveness acceptability curves

## Data Availability

The original data in this study are included in the article and further inquiries can be directed to the corresponding authors.

## Abbreviations

IVF: In vitro fertilization
PGT-A: Preimplantation genetic testing for aneuploidies
PGT-M: Preimplantation genetic testing for monogenic/single gene
PGT-SR: Preimplantation genetic testing for structural chromosome rearrangements
ICSI: Intracytoplasmic sperm injection
CLBR: Cumulative live birth rate
CMR: Cumulative miscarriage rate
ICER: Incremental cost-effectiveness ratio.
