# Supplementary material for: Cost-effectiveness of preimplantation genetic testing for aneuploidy for women with subfertility in China: an economic evaluation using evidence from the CESE-PGS trial
Source: BMC Pregnancy Childbirth. 2023 Apr 14;23:254. doi: 10.1186/s12884-023-05563-z (PMC10103395; doi:10.1186/s12884-023-05563-z)
Supplement: Supplementary file 1 — Additional file 1: eTable 1. Characteristics of the Patients in CESE-PGS trial [file 12884_2023_5563_MOESM1_ESM.docx]

**eTable 1. Characteristics of the Patients in CESE-PGS trial**

| Characteristic | PGT-A Group | Conventional-IVF Group |
| --- | --- | --- |
| Age (year) | 29.1±3.6 | 29.2±3.5 |
| Body-mass index | 23.0±3.4 | 22.9±3.5 |
| Indication for IVF (%) |  |  |
| Ovulatory dysfunction | 6.8 | 5.8 |
| Tubal factor | 51.8 | 56.9 |
| Endometriosis | 0.5 | 0.3 |
| Male factor | 15.2 | 15.5 |
| Combined factors | 20.1 | 17.8 |
| Unexplained | 5.6 | 3.6 |
| Ultrasonographic findings |  |  |
| Antral follicle count in both ovaries | 22.1±10.6 | 21.9±10.0 |
| Endometrial thickness (mm) | 7.3±2.8 | 7.4±2.7 |
| Laboratory testing |  |  |
| Follicle-stimulating hormone （IU/liter） | 6.0±1.6 | 6.1±1.7 |
| Luteinizing hormone (IU/liter) | 6.9±4.8 | 6.7±4.8 |
| Estradiol (pg/ml) | 40.8±25.9 | 41.2±24.0 |
| Total testosterone (ng/ml) | 0.4±0.2 | 0.4±0.2 |
| Prolactin (ng/ml) | 17.6±8.9 | 18.0±9.0 |
