# Supplementary material for: Cost-effectiveness of preimplantation genetic testing for aneuploidy for women with subfertility in China: an economic evaluation using evidence from the CESE-PGS trial
Source: BMC Pregnancy Childbirth. 2023 Apr 14;23:254. doi: 10.1186/s12884-023-05563-z (PMC10103395; doi:10.1186/s12884-023-05563-z)
Supplement: Supplementary file 2 — Additional file 2: eTable 2. Procedures for incorporating cost estimates in IVF [file 12884_2023_5563_MOESM2_ESM.docx]

**eTable 2. Procedures for incorporating cost estimates in IVF**

|  | IVF stage ^a^ | ET stage ^b^ |
| --- | --- | --- |
| Cost of drugs | Controlled Ovarian Hyperstimulation | Endometrial preparation |
|  |  | luteal-phase support |
| Cost of Examination | Laboratory testing | Laboratory testing |
|  | Ultrasonography | Ultrasonography |
| Cost of IVF procedures | Ovum pickup | Cryo-preserved embryo transfer |
|  | IVF laboratory procedure | Thawing procedure |

**Abbreviations:** IVF, In Vitro Fertilization; ET, embryo transfer.

^a^ IVF stage includes all procedures from the patients’ infertility diagnosis to the acquisition of embryos;

^b^ ET stage includes all procedures from embryo transfer to live birth.
