# Supplementary material for: Cost-effectiveness of preimplantation genetic testing for aneuploidy for women with subfertility in China: an economic evaluation using evidence from the CESE-PGS trial
Source: BMC Pregnancy Childbirth. 2023 Apr 14;23:254. doi: 10.1186/s12884-023-05563-z (PMC10103395; doi:10.1186/s12884-023-05563-z)
Supplement: Supplementary file 3 — Additional file 3: eTable 3. Results of probabilistic sensitivity analysis [file 12884_2023_5563_MOESM3_ESM.docx]

**eTable 3. Results of probabilistic sensitivity analysis**

| Parameter | PGT-A | Conventional-IVF | Incremental Cost |
| --- | --- | --- | --- |
| Median costs per patient (CNY) | 34,313.67 | 31,364.33 | 2,949.34 |
| 95% confidence interval Costs per patient (CNY) | 26,835.91-44,949.73 | 24,254.59-42,255.97 | 2,581.32-2,693.77 |
| Cumulative live birth rate | 0.84 | 0.90 | - |
| 95% confidence interval Cumulative live birth rate | 0.82- 0.86 | 0.87- 0.92 | - |
| Cumulative miscarriage rate | 0.08 | 0.15 | - |
| 95% confidence interval Cumulative miscarriage rate | 0.06 - 0.11 | 0.11 - 0.18 | - |

**Abbreviations:** IVF, In Vitro Fertilization; PGT-A, preimplantation genetic testing for aneuploidy.
