# Supplementary material for: Cost-effectiveness of preimplantation genetic testing for aneuploidy for women with subfertility in China: an economic evaluation using evidence from the CESE-PGS trial
Source: BMC Pregnancy Childbirth. 2023 Apr 14;23:254. doi: 10.1186/s12884-023-05563-z (PMC10103395; doi:10.1186/s12884-023-05563-z)
Supplement: Supplementary file 4 — Additional file 4: eFigure 1. Costs per live birth and per patient with and without PGT-A in the base-case [file 12884_2023_5563_MOESM4_ESM.docx]

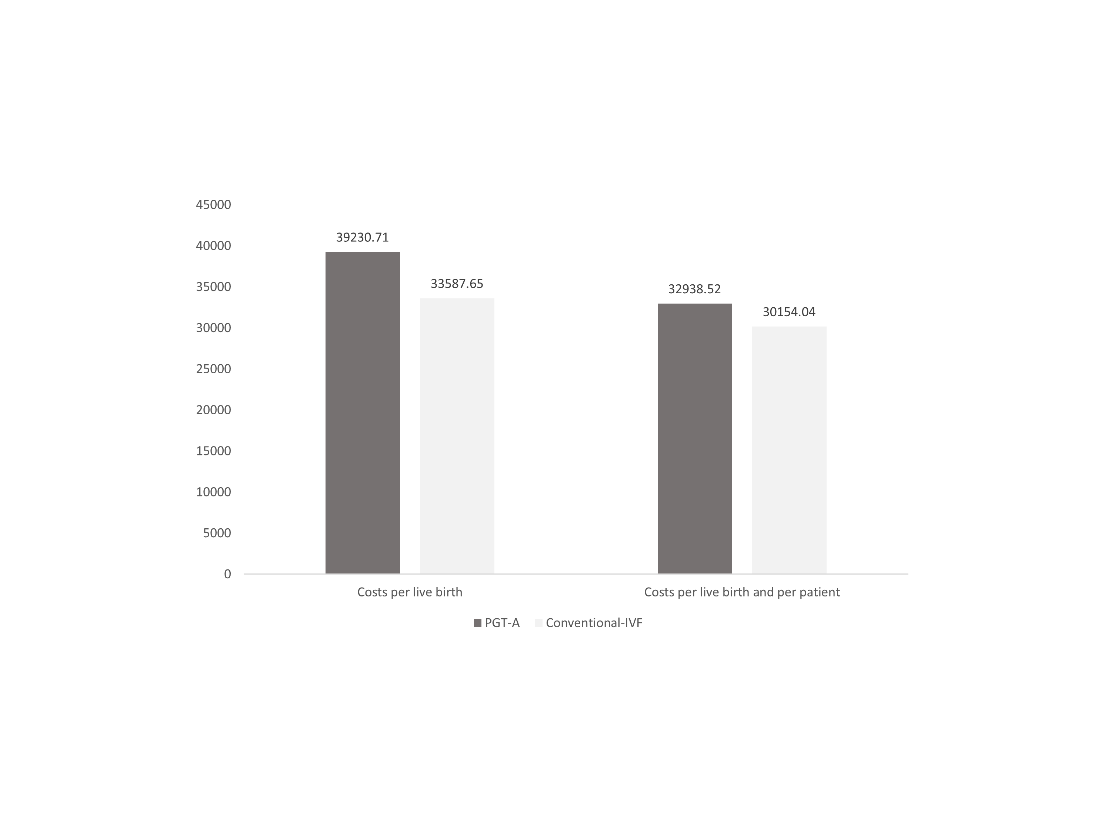


**eFigure 1. Costs per live birth and per patient with and without PGT-A in the base-case**

**Note:** Con IVF, conventional-IVF; PGT-A, preimplantation genetic testing for aneuploidy.
