# Supplementary material for: Cost-effectiveness of preimplantation genetic testing for aneuploidy for women with subfertility in China: an economic evaluation using evidence from the CESE-PGS trial
Source: BMC Pregnancy Childbirth. 2023 Apr 14;23:254. doi: 10.1186/s12884-023-05563-z (PMC10103395; doi:10.1186/s12884-023-05563-z)
Supplement: Supplementary file 5 — Additional file 5: eFigure 2. Cost-effectiveness acceptability curves [file 12884_2023_5563_MOESM5_ESM.docx]

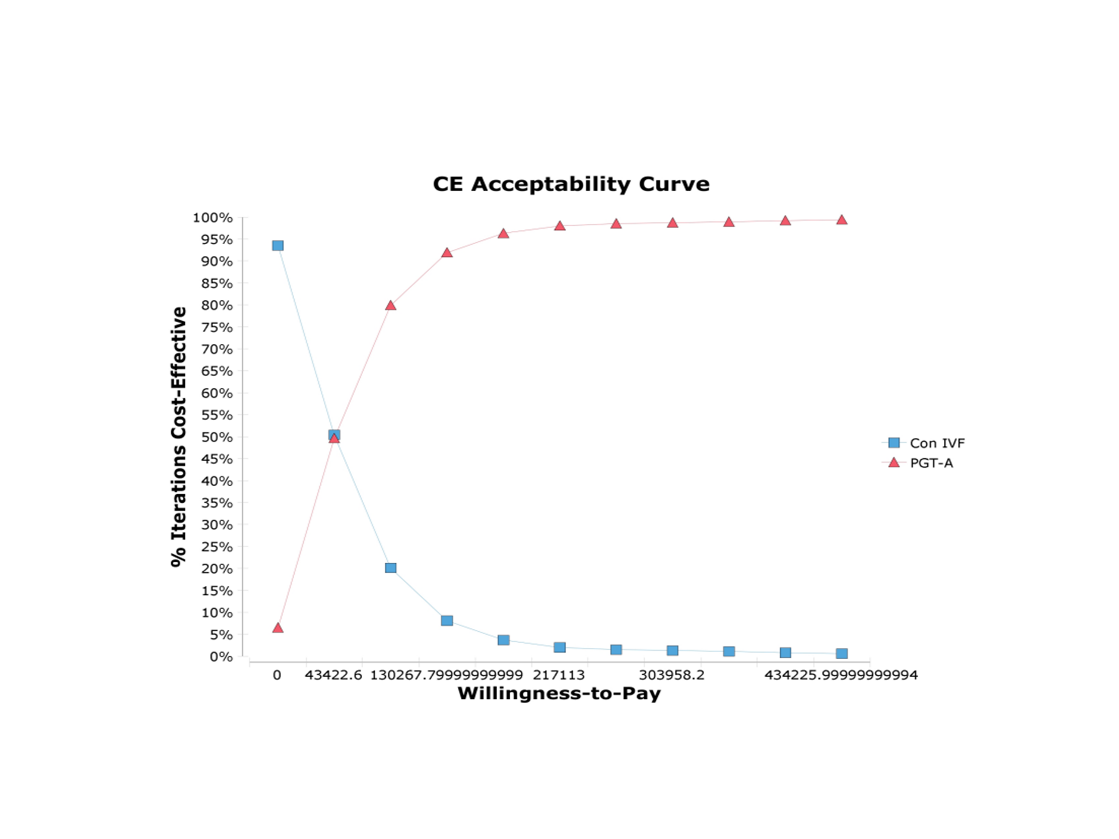


**eFigure 2. Cost-effectiveness acceptability curves**

**Note:** The horizontal axis represents the willingness to pay for miscarriage prevention, as a unit of effectiveness.
